# Antibody kinetics and serologic profiles of SARS-CoV-2 infection using two serologic assays

**DOI:** 10.1371/journal.pone.0240395

**Published:** 2020-10-22

**Authors:** Oh Joo Kweon, Yong Kwan Lim, Hye Ryoun Kim, Min-Chul Kim, Seong-Ho Choi, Jin-Won Chung, Mi-Kyung Lee

**Affiliations:** 1 Department of Laboratory Medicine, Chung-Ang University College of Medicine, Seoul, Republic of Korea; 2 Division of Infectious Diseases, Department of Internal Medicine, Chung-Ang University College of Medicine, Seoul, Republic of Korea; New York State Department of Health, UNITED STATES

## Abstract

**Background:**

Coronavirus disease 2019 (COVID-19) is an emerging threat worldwide. This study aims to assess the serologic profiles and time kinetics of antibodies against severe acute respiratory syndrome coronavirus 2 (SARS-CoV-2) in patients with COVID-19 using two immunoassays.

**Methods:**

A total of 97 samples serially collected from 17 patients with COVID-19 and 137 negative control samples were analyzed for IgM and IgG against SARS-CoV-2 using the AFIAS COVID-19 Ab (Boditech Med Inc., Chuncheon, Republic of Korea) and the EDI^™^ Novel Coronavirus COVID-19 ELISA Kit (Epitope Diagnostics, Inc., San Diego, CA).

**Results:**

With both assays, IgM and IgG rapidly increased after 7 days post symptom onset (PSO). IgM antibody levels reached a peak at 15–35 d PSO and gradually decreased. IgG levels gradually increased and remained at similar levels after 22–35 d. The diagnostic sensitivities of IgM/IgG for ≤14d PSO were 21.4%/35.7~57.1% and increased to 41.2~52.9%/88.2~94.1% at >14 d PSO with specificities of 98.5%/94.2% for AFIAS COVID-19 Ab and 100.0%/96.4% for EDI^™^ Novel Coronavirus COVID-19 ELISA Kit. Among 137 negative controls, 12 samples (8.8%) showed positive or indeterminate results.

**Conclusions:**

The antibody kinetics against SARS-CoV-2 are similar to common findings of acute viral infectious diseases. Antibody testing is useful for ruling out SARS-CoV-2 infection after 14 d PSO, detecting past infection, and epidemiologic surveys.

## Introduction

Coronavirus disease 2019 (COVID-19), the lung disease caused by severe acute respiratory syndrome coronavirus 2 (SARS-CoV-2), is an emerging threat to individual and public health. On March 11, 2020, the World Health Organization (WHO) declared COVID-19 a pandemic [1]. As of June 26, 2020, the number of patients infected with SARS-CoV-2 has exceeded 9,296,202 globally, and more than 479,133 people have died of COVID-19 in 216 countries [1].

Timely and accurate diagnosis of COVID-19 infection is important for appropriate treatment and limiting further spread of the virus [2]. The current gold standard for diagnosing SARS-CoV-2 infection is a molecular test using real-time reverse transcription-polymerase chain reaction (rRT-PCR) with respiratory tract specimens [2, 3]. However, the quality of PCR testing is critical in obtaining accurate results. Diagnostic accuracy of PCR testing is highly dependent on many factors, including sample types, sample collection methods, sample handling, sample transport and storage, presence of interfering substances, manual errors, and variety of targeted genes for diagnosis and primer designs [2, 4]. Obtaining nasopharyngeal or throat swabs is an uncomfortable procedure that can cause coughing and sneezing, which may generate aerosols as a potential hazard for healthcare workers [5].

Serologic testing is emerging as an additional diagnostic tool for COVID-19 [6]. Although the current role of serologic tests remains confined to epidemiologic purposes [7], accurate assessment of immunologic response may provide several benefits, including diagnosis of patients several days after symptom onset, diagnosis of suspected cases with repeatedly negative results by molecular test, identification of individuals who could serve as donors for plasma immunotherapy, determining the immune status of individuals, and monitoring immune responses to candidate COVID-19 vaccine candidates [8].

Understanding the kinetics of the immune response and antibody dynamics against SARS-CoV-2 is the cornerstone for serologic testing. This study reports the time kinetics of IgM and IgG antibodies in COVID-19 and assesses the serologic profiles of patients with COVID-19 using two commercially available serologic assays based on fluorescent immunoassay (FIA) and enzyme-linked immunosorbent assay (ELISA) from a single medical institution in Seoul, Republic of Korea.

## Materials and methods

### Ethics statement

The protocol was approved by the Institutional Review Board (IRB) of Chung-Ang University Hospital (Seoul, Republic of Korea; approval no. 2042-002-412), and informed consent was obtained from all study subjects.

### Patients and samples

From February to June 2020, a total of 17 symptomatic patients with COVID-19 who were positive for SARS-CoV-2 from upper respiratory tract specimens were enrolled in the study from Chung-Ang University Hospital. Of these, a total of 97 serial serum samples (median 5 samples per patient, minimum 4, and maximum 9) were collected, and their serological profiles were assessed. Twelve (70.6%) patients were male, the median age was 62 yrs (minimum 20, and maximum 91), and all patients were Korean except for one Polish patient. Symptoms used for patient selection included upper/lower respiratory symptoms, fatigue, headache, body ache, fever, chills, diarrhea, nausea, vomiting, loss of taste or smell, and/or chest pain [9].

As a negative control, 137 serum samples were obtained from 137 subjects with negative results on SARS-CoV-2 molecular tests with respiratory samples. The median age of the negative control group was 61 yrs, and 73 patients (53.3%) were male. Among 137 patients, 6 (4.4%) patients had a history of CoV other than SARS-CoV-2, such as human CoV (HCoV)-229E, HCoV-NL63, or HCoV-OC43. They were infected with other CoVs several months ago (median 4 months, minimum 2 months, and maximum 8 months).

All serum samples used in this study were remnant samples after analysis for clinical care and were stored at -70°C until analysis.

### Serologic assays

Detection of anti-SARS-CoV-2 antibody IgM/IgG was performed with two serologic assays, AFIAS COVID-19 Ab (Boditech Med Inc., Chuncheon, Republic of Korea), an FIA, and EDI^™^ Novel Coronavirus COVID-19 IgG/IgM ELISA Kit (Epitope Diagnostics, Inc., San Diego, CA).

The AFIAS COVID-19 Ab (hereafter, AFIAS) assay is a sandwich FIA for automatic qualitative/semiquantitative (through signal intensity, cut-off index (COI)) determination of IgG and IgM antibodies separately against SARS-CoV-2 using recombinant nucleocapsid protein as an antigen [10]. This assay is a point-of-care test and reports results within 20 minutes using whole blood, serum, or plasma. For testing, 100 uL of serum was dispensed into the sample well on the cartridge. After loading the cartridge into the AFIAS-6 system (Boditech Med Inc.), all procedures from loading the buffer into the cartridge to obtaining test results were conducted automatically. Briefly, a fluorescence-labeled conjugate in dried detection buffer binds to the antibody in a sample to form antibody-antigen complexes. The complexes migrate onto the nitrocellulose membrane and are captured by immobilized-anti-human IgG and anti-human IgM on the test strip. The presence of more antibodies in the sample results in formation of more antigen-antibody complexes and leads to stronger intensity fluorescence signal on detector antigen, which is processed to determine the relative concentration of anti-SARS-CoV-2 antibodies in the sample [10]. Results were interpreted according to the COI. The COI was determined by an equation based on the sample-to-positive control signal ratio. COI<0.9 was interpreted as negative for IgM/IgG, 0.9≤COI<1.1 was indeterminate, and COI≥1.1 was positive for IgM/IgG.

EDI^™^ Novel Coronavirus COVID-19 IgM ELISA Kit (hereafter, IgM ELISA) and EDI^™^ Novel Coronavirus COVID-19 IgG ELISA Kit (hereafter, IgG ELISA) utilize the microplate-based enzyme immunoassay technique for qualitative/semiquantitative antibody detection [11, 12]. ELISA with the serum samples was performed according to the manufacturer’s instructions. In the assay procedures, “anti-human IgM antibody—human COVID-19 IgM antibody—horseradish peroxidase (HRP) labeled COVID-19 nucleoprotein antigen” and “COVID-19 recombinant antigen—human anti-COVID-19 IgG antibody—HRP labeled anti-human IgG tracer antibody” are formed in the microplate for IgG and IgM tests, respectively. These immunocomplexes were incubated with substrate solution, and the optical densities (ODs) of each well were measured in a spectrophotometric microplate reader. With the OD of negative controls, positive and negative cutoffs of each microplate were calculated according to package inserts. ODs between the negative and positive cut-off were interpreted as indeterminate results. Additionally, the sample-to-positive control OD ratio (S/P ratio) was obtained; S/P ratio higher than 1.0 was interpreted as a positive result.

### Study design and statistical analysis

For time kinetics evaluation, 97 serial samples from 17 symptomatic patients with COVID-19 were assessed following time frames according to the days (d) past symptom onset (PSO); 1–7 d (22 samples from 8 patients), 8–14 d (26 from 14), 15–21 d (20 from 11), 22–35 d (12 from 6), 36–49 d (12 from 8), and ≥50 d (5 from 3).

Dataset was entered into Microsoft Excel (Microsoft, WA, USA) and analyzed using R version 3.6.1 (http://www.R-project.org/). Seropositive rates were calculated according to the time frame, and graphical representation of time kinetic results with smoothing splines was carried out using R version 3.6.1.

To determine the diagnostic accuracy of serologic assays for COVID-19, diagnostic sensitivity and specificities were calculated subdivided by PSO of ≤14 d and >14 d. To determine the concordance of serologic assays, Cohen’s κ value and total agreement percentage were calculated using 3-by-3 crosstab analysis. The κ values were interpreted according to the criteria proposed by Landis & Koch [13] as bad for values of 0.01–0.20, fair for 0.21–0.40, moderate for 0.41–0.60, strong for 0.61–0.80, and almost perfect for 0.81–1.00.

## Results

### Time kinetics of antibodies against SARS-CoV-2

**Fig 1:**
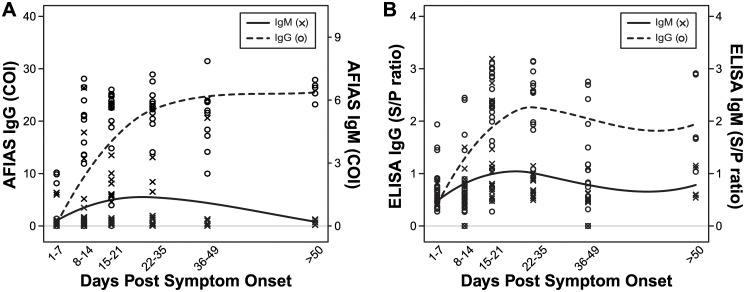
Time kinetics for IgG and IgM antibodies against SARS-CoV-2 of 17 COVID-19 patients according to the days post symptom onset (PSO), using **A**. the AFIAS COVID-19 Ab (Boditech Med Inc., Chuncheon, Republic of Korea) and **B**. EDI^™^ Novel Coronavirus COVID-19 IgM and IgG ELISA Kit (Epitope Diagnostics, Inc., San Diego, CA). From both assays, IgM and IgG rapidly increased after 7 days PSO. IgM antibody levels reached to peak point at 15–35 days PSO and gradually decreased. IgG levels gradually increased and remained at similar degrees after 22–35 days PSO.

Fig 1 shows the kinetic results for patients with COVID-19 at different PSO periods assessed by two serologic assays according to COI and S/P ratio values. The two assays showed similar antibody time kinetics to each other. The smoothing splines of both assays showed that IgM and IgG antibody titer rapidly increased after 7 d PSO. For IgM, the smoothing splines of both assays reached a peak at 15–35 d PSO and then gradually decreased. For IgG, the smoothing splines gradually increased and remained at similar levels at 22–35 d PSO. Detailed results from two serologic assays are provided in S1 Table.

Seropositive rates of patients with COVID-19 according to PSO days are listed in Table 1. IgM seropositive rates before 7 d PSO was 12.5% (1/8) from both assays. Both assays showed the highest IgM seropositive rate in the period of 15–21 d PSO. IgG seropositivity of samples obtained before 7 d PSO was lower than 12.5%, and it reached 100% at 22–35 d PSO. One patient showed negative results on 36–49 d PSO from IgG ELISA, but positivity by AFIAS.

**Table 1:** Seropositive rates of 17 patients with COVID-19 according to days post symptom onset.

Days post symptom onset	IgM		IgG	
	AFIAS	ELISA	AFIAS	ELISA
01-Jul	12.50%	12.50%	12.50%	0.00%
(N = 8)^1^	(1/8)	(1/8)	(1/8)	(0/8)
Aug-14	37.50%	14.30%	57.10%	35.70%
(N = 14)	(3/8)	(2/14)	(8/14)	(5/14)
15–21	54.50%	72.70%	90.90%	90.90%
(N = 11)	(6/11)	(8/11)	(10/11)	(10/11)
22–35	33.50%	33.50%	100.00%	100.00%
(N = 6)	(2/6)	(2/6)	(6/6)	(6/6)
36–49	25.00%	12.50%	100.00%	87.50%
(N = 8)	(2/8)	(1/8)	(8/8)	(7/8)
>50	0.00%	33.30%	100.00%	100.00%
(N = 3)	(0/3)	(1/3)	(3/3)	(3/3)

Abbreviation: COVID-19, coronavirus disease 2019; AFIAS, AFIAS COVID-19 Ab (Boditech Med Inc., Chuncheon, Republic of Korea); ELISA, EDI^™^ Novel Coronavirus COVID-19 IgG/IgM ELISA Kit (Epitope Diagnostics, Inc., San Diego, CA)

^1^Number of patients

### Diagnostic accuracy of serologic assays for COVID-19

The diagnostic accuracy of serologic assays is listed in Tables 2 and 3. For AFIAS, the diagnostic sensitivity of IgM/IgG for ≤14 d PSO was 21.4/57.1% and 41.2/94.1% at >14 d PSO, with specificity of 98.5%/94.2%. For ELISA, the diagnostic sensitivity of IgM/IgG for ≤14 d PSO was 21.4%/35.7% and 52.9%/88.2% at >14 d PSO, with specificity of 100.0%/96.4%.

**Table 2:** Diagnostic performance of AFIAS COVID-19 Ab for COVID-19 according to days post symptom onset with 17 patients and 137 negative controls.

AFIAS COVID-19 Ab^a^		Sensitivity (%)	Specificity (%)
IgM	≤14d^b^	21.4	
		(7.7–47.4)^c^	98.5
	>14d	41.2	(94.5–99.9)
		(21.7–63.9)	
IgG	≤14d	57.1	
		(32.7–78.5)	94.2
	>14d	94.1	(88.7–97.2)
		(73.2–98.8)	
IgG and/or IgM	≤14d	71.4	
		(45.5–88.1)	94.2
	>14d	100	(88.7–97.2)
		(81.8–99.8)	

Abbreviation: COVID-19, coronavirus disease 2019

Diagnosis of COVID-19 was made by real-time reverse transcriptase polymerase chain reaction with respiratory specimens

^a^Boditech Med Inc., Chuncheon, Republic of Korea

^b^Days post symptom onset

^c^95% confidence interval

**Table 3:** Diagnostic performance of EDI^™^ ELISA kits for COVID-19 according to days post symptom onset with 17 symptomatic patients and 137 negative controls.

ELISA^a^		Sensitivity (%)	Specificity (%)
IgM	≤14d^b^	21.4	
		(7.7–47.4)^c^	100
	>14d	52.9	(96.7–100.0)
		(31.1–73.7)	
IgG	≤14d	35.7	
		(16.5–61.1)	96.4
	>14d	88.2	(91.5–98.7)
		(65.8–96.6)	
IgG and/or IgM	≤14d	50	
		(26.9–73.1)	99.3
	>14d	88.2	(95.6–100.0)
		(65.8–96.6)	

Abbreviation: COVID-19, coronavirus disease 2019

Diagnosis of COVID-19 was made by real-time reverse transcriptase polymerase chain reaction with respiratory specimens

^a^ EDI^™^ Novel Coronavirus COVID-19 IgG/IgM ELISA Kit (Epitope Diagnostics, Inc., San Diego, CA)

^b^Days post symptom onset

^c^95% confidence interval

For the 137 negative controls, 12 (8.8%) samples showed positive or indeterminate results from any of the two serologic assays. The details of their antibody profiles are listed in Table 4. For IgM, 2 samples (1.5%) showed positive or indeterminate results by AFIAS, and they were all negative by ELISA. For IgG, 9 (6.6%) samples showed positive or indeterminate results. Among them, 1 sample (0.7%) was positive by both assays.

**Table 4:** Serologic profiles of 12 negative controls with positive or indeterminate results from AFIAS COVID-19 Ab and/or EDI^™^ Novel Coronavirus COVID-19 IgG/IgM ELISA Kit among 137 total normal controls.

Non- COVID-19	IgM		IgG	
Patients No.	AFIAS	ELISA	AFIAS	ELISA
1	Indeterminate	Negative	Negative	Negative
	(1.08) ^a^	(0.64)	(0.00)	(0.35)
2	Positive	Negative	Negative	Negative
	(1.42)	(0.48)	(0.00)	(0.74)
3	Negative	Negative	Indeterminate	Negative
	(0.00)	(0.47)	(0.94)	(0.58)
4	Negative	Negative	Positive	Negative
	(0.00)	(0.50)	(10.25)	(0.59)
5	Negative	Negative	Positive	Negative
	(0.01)	(0.69)	(14.17)	(0.54)
6	Negative	Negative	Positive	Negative
	(0.00)	(0.50)	(6.50)	(0.49)
7	Negative	Negative	Positive	Negative
	(0.00)	(0.47)	(26.04)	(0.47)
8	Negative	Negative	Positive	Positive
	(0.00)	(0.51)	(15.58)	(1.43)
9	Negative	Negative	Positive	Indeterminate
	(0.00)	(0.46)	(20.87)	(0.89)
10	Negative	Negative	Positive	Indeterminate
	(0.00)	(0.54)	(4.78)	(0.82)
11	Negative	Negative	Negative	Indeterminate
	(0.25)	(0.53)	(0.00)	(0.96)
12	Negative	Negative	Negative	Indeterminate
	(0.00)	(0.48)	(0.00)	(0.99)

Abbreviation: COVID-19, coronavirus disease 2019; AFIAS, AFIAS COVID-19 Ab; ELISA, EDI^™^ Novel Coronavirus COVID-19 IgG/IgM ELISA Kit Ab

^a^ Cut-Off Index for AFIAS, Sample-to-positive control optical density ratio for ELISA

Among 6 negative controls with past medical history of CoV other than SARS-CoV-2 infection, there was no seropositivity or cross-reactivity in IgM or IgG with any assay.

### Concordance between two serologic assays

The concordance between serologic assays was assessed. For IgM, κ value was 0.40 (0.25–0.55, fair agreement) with a total agreement rate of 87.0% (82.2%-90.7%). For IgG, the κ value was 0.75 (0.67–0.84, strong agreement) with a total agreement rate of 89.8% (85.4%-93.1%).

## Discussion

A beta-coronavirus, SARS-CoV-2 is the seventh member of the *Coronaviridae* family of viruses and is the causative agent of COVID-19 in humans [14]. Molecular testing of respiratory tract samples to detect SARS-CoV-2 RNA remains the preferred diagnostic test for patients with COVID-19. However, interest in using serologic assays to detect antibodies against SARS-CoV-2 is also increasing [8]. Unlike molecular testing, serologic assays are indirect viral testing, and it is important to understand the kinetics of the immune response against SARS-CoV-2. Currently, serologic assays targeting COVID-19 are highly variable in their assay principles or formats, which include ELISA, lateral flow immunoassays, chemiluminescent immunoassays (CLIA), and microsphere immunoassays [2, 8, 15–20]. They also differ in the SARS-CoV-2 antigens used, such as recombinant nucleocapsid protein or spike protein [8]. Furthermore, there is insufficient performance characteristics data for each assay and lack of commercially accessible reference tests. Rigorous verification study with serially collected serum from COVID-19 patients is required to ensure analytic performance and clinical accuracy.

The observed antibody kinetics against SARS-CoV-2 were consistent with common findings of acute viral infectious diseases [21, 22]. Time kinetics results showed that most signal intensities (COI for AFIAS, S/P ratio for ELISA) of IgM and IgG from both tests were below the cut-off at 1–7 d PSO. After that, the signal values of IgM and IgG from both tests increased rapidly. The COI of IgG continued to increase until 22–35 d PSO and was maintained at similar levels thereafter. The COI of IgM reached a peak at 15–35 d PSO and then gradually decreased. The patterns of IgG and IgM titer kinetics were also similar to those of previous studies of COVID-19 [2, 15, 20, 23–25]. However, the timing of IgM seroconversion varies among published studies. Among 8 patients at ≤7 d PSO in the present study, only 1 patient (12.5%) showed positive results by both assays. This was discordant to several other studies in which a significant number of patients showed IgM positivity in this period. Guo et al reported that IgM antibodies were detectable in 85.4% (35/41) of COVID-19 patients before 7 d PSO [23], and Xiang et al. reported 47.4% (9/19) IgM positivity with samples obtained before 9 d PSO [15]. Using CLIA, Padoan et al. also reported 58.3% IgM positivity before 9 d PSO (7/12) [2]. Studies concordant to our study also exist. Tan et al. reported that IgM can be detected in patient samples at 7 days PSO in only 10.3% of patients [24], Tuaillon et al. revealed that samples collected during the first six days of COVID-19 symptoms were rarely reported using six commercial point-of-care tests [20], and Yang et al. concluded that values were lower than 15% using cyclic enhanced fluorescence assay [25]. Those discordances may stem from differences in severity, antibody detecting methodology, and/or definition of the date of first symptom onset. Until now, the number of studies investigating time of IgM seroconversion is limited. Further studies with various assay techniques and large subject numbers are needed to elucidate this discordance.

The sensitivities of IgM antibody detection were as low as 21.4% (same in both assays) in the samples collected ≤14 d PSO and 41.2%~52.9% in samples >14 d PSO. These findings indicated that in patients infected with SARS-CoV-2, IgM seroconversion may not develop or might not be detected until the middle or late stages of infection. In other words, SARS-CoV-2 infection may be missed based on IgM seropositivity; thus, IgM tests must not be solely used in COVID-19 diagnosis and should be used only as a supportive tool in addition to molecular tests. In addition, although the specificities of IgM tests were satisfactory (above 98.5% in this study), IgM titers in patients with SARS-CoV-2 infection showed a significant reduction after 35 d PSO, their utility in detecting past infection or epidemiologic studies is limited. Taken together, we did not observe any benefit of using IgM testing for COVID-19.

The sensitivity of IgG samples from ≤14 d PSO was as low as 35.7%~57.1%, but it sharply increased for >14 d PSO to 88.2%~94.1%. This means that almost all patients with COVID-19 showed seroconversion after 14 d PSO, and IgG seronegative subjects in this period are considered less likely to be infected with SARS-CoV-2. In addition, both assays showed 94.2~96.4% of IgG specificities and increased IgG titers in COVID-19 patients were maintained. Thus IgG serologic assays can be useful for ruling out SARS-CoV-2 infection after 14 d PSO, detecting past infection, and epidemiologic surveys. Nevertheless, caution is always needed for immunocompromised patients or those with mild symptoms after SARS-CoV-2 infection because their antibody titers may be lower than those of other patients [26, 27].

In the present study, 7.3% (10 of 137) of the negative control group showed IgG (10 samples, 7.3%) seropositivity from one or both assays; 5 samples were from AFIAS, 2 samples were from ELISA, and 3 samples were from both assays. The possible causes of IgG positivity in non-COVID-19 patients include non-specific reaction by serum substances or true positivity. The former can be resolved by assessing samples with other serologic assays, using different antigens to detect antibodies, and the latter implies that subjects had past asymptomatic infections that are currently not vigorously investigated. Xiang et al. reported that 5% (3/60) of the negative control group showed IgG seropositivity [15], similar to our results. One concern regarding SARS-CoV-2 serologic assay specificity is potential cross-reactivity with antibodies to common circulating CoVs, including HCoV-229E, HCoV-NL63, HCoV-OC43, or HKU1 [8]. Among 6 non-COVID 19 patients in this study who had past infection history of other HCoVs, no patient showed seropositivity by both assays. However, our study included a small number of patients who had history of other common HCoV. Further investigation of cross-reactivity to common HCoV is needed using a large number of study subjects and various assays.

In this study, we did not report the PPV and NPV. The PPV and NPV of a test depend heavily on the prevalence of what that test is intended to detect; however, we do not currently know the exact prevalence of SARS-CoV-2 antibody positive-individuals in the community, and the prevalence may change based on the duration [28]. A prevalence based on molecular tests may be helpful but may also be inaccurate or underestimated [29]; this is because all suspected patients are not tested, and asymptomatic infection may also be present. Assuming a prevalence of 0.1% for AFIAS, the estimated PPVs of IgM/IgG for ≤14 d PSO and >14 d PSO were 1.4%/1.0% and 2.7%/1.6%, respectively, with estimated NPVs of 99.9%/100% and 99.9%/100%, respectively. For ELISA, the estimated PPVs of IgM/IgG for ≤14 d PSO and >14 d PSO were 100%/1.0% and 100%/2.4%, respectively, with estimated NPVs of 99.9%/99.9% and 100%/100%, respectively. Nevertheless, these are estimated values, and the exact PPV and NPV must be calculated with an appropriate prevalence for each community.

AFIAS, the point-of-care FIA test, has several advantages compared to other assays. The assay time for antibody analysis is relatively faster (less than 20 min.) than other assays, and the analysis procedure is simpler especially compared to ELISA format tests [15, 24]. It also has relatively simple requirements. An automated fluorescent immunoassay system is utilized for analysis. The point-of-care bench-top analyzer is relatively compact compared to other automated assays. For these reasons, AFIAS COVID-19 Ab could be useful, especially in resource-limited settings.

This study has several limitations. First, the relatively small number of patients with COVID-19 and their sample sizes could have affected the study outcomes. The small sample size of the negative control group could have also affected the specificities of the assays. Second, antibody detection with another reliable validated method was not available for comparison studies. Because the analytical performances of immunoassays vary, another serologic assay would be helpful to interpret disagreement results between AFIAS and ELISA. Finally, because the number of days PSO was highly dependent on patient memory, it is likely inaccurate to within a few days.

In conclusion, we demonstrated that IgM and IgG antibodies against SARS-CoV-2 in patients with COVID-19 were mainly detected after 14 d PSO, IgM levels reached a peak level at 15–35 d PSO and gradually decreased, and IgG levels reached a peak level and remained at similar levels after 22–35 d PSO. Testing for antibodies against SARS-CoV-2, especially IgG, has the potential for ruling out SARS-CoV-2 infection after 14 d PSO, detecting past infection, and epidemiologic surveys.

## Supporting information

S1 Table Details of serologic test results from AFIAS COVID-19 Ab and/or EDI^™^ Novel Coronavirus COVID-19 IgG/IgM ELISA Kit for 17 symptomatic COVID-19 patients. (PDF)

## References

[1] World Health Organization. Coronavirus disease (COVID-19) Pandemic: World Health Organization; 2020 [cited 26 June 2020]. In: WHO website [Internet]. Geneva, Switzerland. https://www.who.int/emergencies/diseases/novel-coronavirus-2019.

[2] Padoan A, Cosma C, Sciacovelli L, Faggian D, Plebani M. Analytical performances of a chemiluminescence immunoassay for SARS-CoV-2 IgM/IgG and antibody kinetics. Clinical chemistry and laboratory medicine. 2020 Epub 2020/04/18. 10.1515/cclm-2020-0443 . 32301749

[3] Jin YH, Cai L, Cheng ZS, Cheng H, Deng T, Fan YP, et al A rapid advice guideline for the diagnosis and treatment of 2019 novel coronavirus (2019-nCoV) infected pneumonia (standard version). Military Medical Research. 2020;7(1):4 Epub 2020/02/08. 10.1186/s40779-020-0233-6 . 32029004 PMC7003341

[4] Lippi G, Simundic AM, Plebani M. Potential preanalytical and analytical vulnerabilities in the laboratory diagnosis of coronavirus disease 2019 (COVID-19). Clinical chemistry and laboratory medicine. 2020 Epub 2020/03/17. 10.1515/cclm-2020-0285 . 32172228

[5] To KK, Tsang OT, Leung WS, Tam AR, Wu TC, Lung DC, et al Temporal profiles of viral load in posterior oropharyngeal saliva samples and serum antibody responses during infection by SARS-CoV-2: an observational cohort study. The Lancet Infectious diseases. 2020 Epub 2020/03/28. 10.1016/s1473-3099(20)30196-1 . 32213337 PMC7158907

[6] Lippi G, Salvagno GL, Pegoraro M, Militello V, Caloi C, Peretti A, et al Assessment of immune response to SARS-CoV-2 with fully automated MAGLUMI 2019-nCoV IgG and IgM chemiluminescence immunoassays. Clinical chemistry and laboratory medicine. 2020 Epub 2020/04/18. 10.1515/cclm-2020-0473 . 32301750

[7] Centers for Disease Control and Prevention. COVID-19 Serology Surveillance Strategy [cited 26 June 2020]. In: CDC website [Internet]. Atlanta, GA. https://www.cdc.gov/coronavirus/2019-ncov/covid-data/serology-surveillance/index.html.

[8] Theel ES, Slev P, Wheeler S, Couturier MR, Wong SJ, Kadkhoda K. The Role of Antibody Testing for SARS-CoV-2: Is There One? Journal of clinical microbiology. 2020 Epub 2020/05/01. 10.1128/jcm.00797-20 . 32350047 PMC7383527

[9] Guan W-j, Ni Z-y, Hu Y, Liang W-h, Ou C-q, He J-x, et al Clinical Characteristics of Coronavirus Disease 2019 in China. New England Journal of Medicine. 2020;382(18):1708–20. 10.1056/NEJMoa2002032 32109013 PMC7092819

[10] Boditech Med Inc., AFIAS COVID-19 Ab Insert Paper, Rev. 00. April 8, 2020.

[11] Epitope Diagnostics Inc., EDI Novel Coronavirus COVID-19 IgM ELISA Kit Insert Paper. In: Epitope Diagnostics I, editor. V6 ed.March, 2020.

[12] Epitope Diagnostics Inc., EDI Novel Coronavirus COVID-19 IgG ELISA Kit Insert Paper. EDI Novel Coronavirus COVID-19 IgG ELISA Kit Insert Paper. V6 ed.March 2020.

[13] Landis JR, Koch GG. The measurement of observer agreement for categorical data. Biometrics. 1977;33(1):159–74. Epub 1977/03/01. . 843571

[14] The species Severe acute respiratory syndrome-related coronavirus: classifying 2019-nCoV and naming it SARS-CoV-2. Nature microbiology. 2020;5(4):536–44. Epub 2020/03/04. 10.1038/s41564-020-0695-z . 32123347 PMC7095448

[15] Xiang F, Wang X, He X, Peng Z, Yang B, Zhang J, et al Antibody Detection and Dynamic Characteristics in Patients with COVID-19. Clinical infectious diseases: an official publication of the Infectious Diseases Society of America. 2020 Epub 2020/04/20. 10.1093/cid/ciaa461 . 32306047 PMC7188146

[16] Chen Z, Zhang Z, Zhai X, Li Y, Lin L, Zhao H, et al Rapid and sensitive detection of anti-SARS-CoV-2 IgG using lanthanide-doped nanoparticles-based lateral flow immunoassay. Analytical chemistry. 2020 Epub 2020/04/24. 10.1021/acs.analchem.0c00784 . 32323974

[17] Sun B, Feng Y, Mo X, Zheng P, Wang Q, Li P, et al Kinetics of SARS-CoV-2 specific IgM and IgG responses in COVID-19 patients. Emerging microbes & infections. 2020;9(1):940–8. Epub 2020/05/03. 10.1080/22221751.2020.1762515 . 32357808 PMC7273175

[18] Demey B, Daher N, François C, Lanoix JP, Duverlie G, Castelain S, et al Dynamic profile for the detection of anti-SARS-CoV-2 antibodies using four immunochromatographic assays. The Journal of infection. 2020 Epub 2020/05/12. 10.1016/j.jinf.2020.04.033 . 32389784 PMC7204722

[19] Cheng MP, Yansouni CP, Basta NE, Desjardins M, Kanjilal S, Paquette K, et al Serodiagnostics for Severe Acute Respiratory Syndrome-Related Coronavirus-2: A Narrative Review. Annals of internal medicine. 2020 Epub 2020/06/05. 10.7326/m20-2854 . 32496919 PMC7281623

[20] Tuaillon E, Bolloré K, Pisoni A, Debiesse S, Renault C, Marie S, et al Detection of SARS-CoV-2 antibodies using commercial assays and seroconversion patterns in hospitalized patients. The Journal of infection. 2020 Epub 2020/06/07. 10.1016/j.jinf.2020.05.077 . 32504735 PMC7834649

[21] Chen W, Xu Z, Mu J, Yang L, Gan H, Mu F, et al Antibody response and viraemia during the course of severe acute respiratory syndrome (SARS)-associated coronavirus infection. Journal of medical microbiology. 2004;53(Pt 5):435–8. Epub 2004/04/21. 10.1099/jmm.0.45561-0 . 15096554

[22] Goncalves A, Peeling RW, Chu MC, Gubler DJ, de Silva AM, Harris E, et al Innovative and New Approaches to Laboratory Diagnosis of Zika and Dengue: A Meeting Report. The Journal of infectious diseases. 2018;217(7):1060–8. Epub 2018/01/03. 10.1093/infdis/jix678 . 29294035 PMC6279137

[23] Guo L, Ren L, Yang S, Xiao M, Chang Yang F, et al Profiling Early Humoral Response to Diagnose Novel Coronavirus Disease (COVID-19). Clinical infectious diseases: an official publication of the Infectious Diseases Society of America. 2020 Epub 2020/03/22. 10.1093/cid/ciaa310 . 32198501 PMC7184472

[24] Tan W, Lu Y, Zhang J, Wang J, Dan Y, Tan Z, et al Viral Kinetics and Antibody Responses in Patients with COVID-19. medRxiv. 2020:2020.03.24.20042382. 10.1101/2020.03.24.20042382

[25] Yang HS, Racine-Brzostek SE, Lee WT, Hunt D, Yee J, Chen Z, et al SARS-CoV-2 antibody characterization in emergency department, hospitalized and convalescent patients by two semi-quantitative immunoassays. Clinica chimica acta; international journal of clinical chemistry. 2020;509:117–25. Epub 2020/06/09. 10.1016/j.cca.2020.06.004 . 32505774 PMC7272145

[26] Long Q-X, Tang X-J, Shi Q-L, Li Q, Deng H-J, Yuan J, et al Clinical and immunological assessment of asymptomatic SARS-CoV-2 infections. Nature Medicine. 2020;26(8):1200–4. 10.1038/s41591-020-0965-6 32555424

[27] Kopel E, Oren G, Sidi Y, David D. Inadequate antibody response to rabies vaccine in immunocompromised patient. Emerg Infect Dis. 2012;18(9):1493–5. 10.3201/eid1809.111833 . 22932226 PMC3437714

[28] U.S. Food and Drug Administration. EUA Authorized Serology Test Performance: U.S. Food and Drug Administration; 2020 [cited 2020 August 20]. In: FDA website [Internet]. Silver Spring, MD. https://www.fda.gov/medical-devices/coronavirus-disease-2019-covid-19-emergency-use-authorizations-medical-devices/eua-authorized-serology-test-performance.

[29] Tanne JH. Covid-19: US cases are greatly underestimated, seroprevalence studies suggest. BMJ. 2020;370:m2988 10.1136/bmj.m2988 32709608

