# Supplementary material for: Antibody kinetics and serologic profiles of SARS-CoV-2 infection using two serologic assays
Source: PLoS One. 2020 Oct 22;15(10):e0240395. doi: 10.1371/journal.pone.0240395 (PMC7580996; doi:10.1371/journal.pone.0240395)
Supplement: S1 Table — (PDF) [file pone.0240395.s001.pdf]

**S1 Table. Details of serologic test results from AFIAS COVID-19 Ab and/or EDI™ Novel Coronavirus COVID-19 IgG/IgM ELISA Kit for 17 symptomatic COVID-19 patients**

| COVID-19 Patients | d PSO (d) | IgM | | IgG | |
|---|---|---|---|---|---|
| | | AFIAS (COI) | ELISA (S/P ratio) | AFIAS (COI) | ELISA (S/P ratio) |
| A | 13 | 0.13 | 0.89 | 20.94 | 1.74 |
| | 19 | 0.04 | 0.56 | 24.72 | 2.61 |
| | 23 | 0.02 | 0.57 | 23.77 | 2.54 |
| | 27 | 0.00 | 0.53 | 22.26 | 2.56 |
| | 42 | 0.00 | 0.52 | 23.74 | 2.42 |
| B | 8 | 0.00 | 0.47 | 0.00 | 0.35 |
| | 11 | 0.31 | 1.09 | 0.00 | 0.62 |
| | 15 | 0.38 | 1.10 | 23.20 | 2.26 |
| | 20 | 0.29 | 1.11 | 22.71 | 1.99 |
| | 24 | 0.40 | 0.89 | 22.90 | 1.94 |
| | 27 | 0.31 | 0.66 | 21.61 | 1.83 |
| | 31 | 0.49 | 0.63 | 14.09 | 1.98 |
| | 53 | 0.17 | 0.60 | 26.38 | 1.69 |
| | 67 | 0.04 | 0.59 | 23.20 | 1.66 |
| C | 5 | 0.00 | 0.61 | 0.00 | 0.35 |
| | 8 | 0.00 | 0.48 | 0.00 | 0.29 |
| | 9 | 0.00 | 0.46 | 0.00 | 0.28 |
| | 12 | 0.00 | 0.48 | 0.00 | 0.28 |
| | 38 | 0.00 | 0.50 | 22.03 | 1.95 |
| D | 16 | 1.15 | 1.46 | 14.82 | 3.02 |
| | 20 | 1.47 | 1.04 | 18.82 | 3.10 |
| | 22 | 1.63 | 1.00 | 20.46 | 3.15 |
| | 30 | 2.10 | 0.86 | 23.34 | 3.13 |
| E | 11 | 0.00 | 0.51 | 0.00 | 0.46 |
| | 12 | 0.02 | 0.59 | 0.00 | 0.44 |
| | 14 | 0.13 | 0.78 | 0.00 | 0.77 |
| | 16 | 0.11 | 0.52 | 0.00 | 0.48 |
| | 19 | 0.16 | 1.04 | 20.04 | 2.38 |
| | 24 | 0.06 | 0.92 | 22.72 | 2.73 |
| | 29 | 0.14 | 0.96 | 28.92 | 2.68 |
| | 32 | 0.10 | 1.15 | 24.95 | 2.58 |

***(S1 Table continue)***

| COVID-19 Patients | d PSO (d) | IgM | | IgG | |
|---|---|---|---|---|---|
| | | AFIAS (COI) | ELISA (S/P ratio) | AFIAS (COI) | ELISA (S/P ratio) |
| F | 9 | 0.05 | 0.48 | 0.00 | 0.72 |
| | 11 | 0.20 | 0.58 | 15.93 | 1.45 |
| | 15 | 2.21 | 0.81 | 26.03 | 2.92 |
| | 16 | 2.51 | 0.78 | 23.88 | 3.00 |
| | 17 | 2.02 | 0.70 | 22.78 | 2.85 |
| | 18 | 3.37 | 0.71 | 22.80 | 2.91 |
| | 23 | 3.27 | 0.68 | 19.48 | 2.95 |
| | 43 | 5.15 | 0.23 | 18.39 | 2.75 |
| G | 45 | 0.26 | 0.48 | 9.99 | 1.18 |
| | 47 | 0.33 | 1.10 | 23.73 | 2.69 |
| | 53 | 0.32 | 1.15 | 26.82 | 2.89 |
| | 60 | 0.28 | 1.11 | 25.30 | 2.91 |
| H | 8 | 0.07 | 0.41 | 0.00 | 0.57 |
| | 13 | 4.46 | 0.82 | 20.29 | 2.45 |
| | 14 | 3.21 | 0.78 | 21.20 | 2.41 |
| | 36 | 6.03 | 0.00 | 23.66 | 0.00 |
| I | 8 | 0.01 | 0.38 | 0.00 | 0.27 |
| | 13 | 6.59 | 1.50 | 0.00 | 0.76 |
| | 16 | 5.77 | 3.19 | 0.00 | 2.18 |
| | 17 | 5.66 | 2.73 | 0.00 | 2.08 |
| | 20 | 4.87 | 1.29 | 3.96 | 2.29 |
| J | 3 | 0.14 | 0.42 | 0.00 | 0.48 |
| | 4 | 0.17 | 0.44 | 0.00 | 0.46 |
| | 6 | 0.21 | 0.48 | 0.00 | 0.45 |
| | 9 | 0.15 | 0.47 | 0.00 | 0.52 |
| | 13 | 0.11 | 0.50 | 13.52 | 0.63 |
| | 42 | 0.11 | 0.17 | 14.14 | 0.82 |

*(S1 Table continue)*

| COVID-19 Patients | d PSO (d) | IgM | | IgG | |
|---|---|---|---|---|---|
| | | AFIAS (COI) | ELISA (S/P ratio) | AFIAS (COI) | ELISA (S/P ratio) |
| K | 4 | 0.29 | 0.50 | 0.00 | 0.36 |
| | 5 | 0.29 | 0.53 | 0.00 | 0.36 |
| | 7 | 0.30 | 0.50 | 0.00 | 0.33 |
| | 10 | 0.25 | 0.42 | 0.00 | 0.42 |
| | 14 | 0.15 | 0.50 | 28.11 | 0.89 |
| | 28 | 0.06 | 0.49 | 27.59 | 1.09 |
| | 43 | 0.00 | 0.31 | 31.46 | 1.50 |
| L | 3 | 1.59 | 0.42 | 0.00 | 0.28 |
| | 5 | 1.49 | 0.38 | 0.00 | 0.28 |
| | 12 | 1.29 | 0.00 | 0.00 | 0.00 |
| | 16 | 1.52 | 0.48 | 0.00 | 0.28 |
| M | 40 | 0.00 | 0.45 | 17.21 | 0.32 |
| | 41 | 0.00 | 0.62 | 23.64 | 0.74 |
| | 42 | 0.01 | 0.62 | 23.93 | 0.83 |
| | 44 | 0.09 | 0.63 | 21.59 | 1.07 |
| | 55 | 0.05 | 0.34 | 27.89 | 1.03 |
| N | 5 | 0.06 | 0.56 | 0.00 | 0.91 |
| | 6 | 0.04 | 0.55 | 0.00 | 1.45 |
| | 9 | 0.04 | 0.53 | 0.00 | 0.74 |
| | 12 | 0.37 | 0.58 | 11.88 | 0.63 |
| | 14 | 0.43 | 0.54 | 23.97 | 0.64 |
| | 15 | 0.30 | 0.66 | 22.54 | 1.09 |
| | 19 | 0.26 | 0.26 | 24.94 | 1.53 |
| O | 3 | 0.00 | 0.81 | 0.00 | 1.50 |
| | 4 | 0.00 | 0.89 | 0.00 | 1.94 |
| | 5 | 0.00 | 0.52 | 0.00 | 0.76 |
| | 9 | 0.01 | 0.57 | 0.00 | 0.79 |
| | 16 | 1.14 | 2.38 | 23.42 | 2.83 |

***(S1 Table continue)***

| COVID-19 Patients | d PSO (d) | IgM | | IgG | |
|---|---|---|---|---|---|
| | | AFIAS (COI) | ELISA (S/P ratio) | AFIAS (COI) | ELISA (S/P ratio) |
| P | 3 | 0.06 | 0.60 | 0.00 | 0.73 |
| | 4 | 0.04 | 0.62 | 0.00 | 0.89 |
| | 5 | 0.04 | 0.55 | 0.00 | 0.48 |
| | 9 | 0.24 | 0.54 | 13.63 | 0.51 |
| | 16 | 0.22 | 1.19 | 25.19 | 1.62 |
| Q | 2 | 0.13 | 0.53 | 9.85 | 0.47 |
| | 3 | 0.10 | 0.69 | 8.16 | 0.72 |
| | 4 | 0.10 | 0.51 | 10.20 | 0.69 |
| | 5 | 0.11 | 0.52 | 1.41 | 0.65 |
| | 9 | 0.87 | 0.57 | 26.49 | 0.69 |
| | 16 | 1.40 | 2.39 | 24.84 | 2.36 |

Abbreviation: COVID-19, coronavirus disease 2019; d PSO, days post symptom onset; AFIAS, AFIAS COVID-19 Ab; ELISA, EDI$^{TM}$ Novel Coronavirus COVID-19 IgG/IgM ELISA Kit Ab; COI, cut-off index; S/P ratio, sample-to-positive control optical densities ratio.
